# Preclinical approaches in regenerative medicine for treating end-stage renal disease: a scoping review

**DOI:** 10.3389/fendo.2025.1549744

**Published:** 2025-07-18

**Authors:** Andrea Vigezzi, Paola Brani, Daniela Dalla Gasperina, Lorenzo Azzi, Cristina Giaroni, Davide Inversini, Giuseppe Pettinato, Matthew Massaad, Giulio Carcano, Giuseppe Ietto, Andreina Baj

**Affiliations:** ^1^ Department of Medicine and Technological Innovation, University of Insubria, Varese, Italy; ^2^ Division of Gastroenterology, Department of Medicine, Beth Israel Deaconess Medical Center, Harvard Medical School, Boston, MA, United States

**Keywords:** bioengineering, kidney, organoids, regenerative, stem

## Abstract

A scoping review was conducted to systematically assess the current evidence and emerging applications of regenerative medicine in the treatment of End Stage Renal Disease (ERSD), aiming to map existing knowledge and identify key research gaps. ESRD represents a major global health burden, and despite being managed primarily through dialysis and kidney transplantation, both approaches are limited by morbidity, mortality, and organ donor shortages. Regenerative medicine emerged as a promising alternative, leveraging stem cells technologies and tissue engineering to develop functional renal tissues with the potential to restore or replace damaged kidney structures. Our review protocol was developed in accordance with the PRISMA-ScR guidelines and was prospectively registered on Figshare. Inclusion criteria comprised peer-reviewed articles published between 2004 and 2024, focusing on bioengineering strategies relevant to kidney regeneration and ESRD, with no restrictions on language or geographic origin. Editorials, letters, and non–peer-reviewed sources were excluded. A comprehensive literature search was performed in Medline, Scopus, and Web of Science using pre-defined search terms. Screening and selection were conducted independently by four reviewers working in pairs selected with discrepancies resolved through consensus. A standardized data extraction form was iteratively developed and piloted to collect relevant information on study characteristics and experimental models. Of the 5,869 records initially identified, 111 studies met the inclusion criteria. The findings underscore the therapeutic potential of regenerative medicine in ESRD, with kidney organoids and organ-on-a-chip platforms representing two of the most advanced and translationally relevant approaches currently under investigation. These technologies are increasingly recognized not only for their role in disease modeling and drug screening, but also as potential precursors to fully bioengineered renal replacement therapies.

## Introduction

Chronic Kidney disease (CKD) represents a growing global health crisis, marked by the progressive and irreversible loss of renal function. In its most advanced stage, End Stage Renal Disease (ESRD)., Renal Replacement Treatment (RRT) becomes essential for survival. As of 2017, CKD affects approximately 9.1% of the global population, reflecting a nearly 30% increase from 1990 (1, 2).

Dialysis, while life-sustaining is inherently palliative and fails to address the underlying mechanisms of kidney dysfunction. It is associated with a high burding of comorbidities, including cardiovascular complications, heightened infection risk, and reduced quality of life. Additionally, a critical shortage of compatible donor organs continues to limit access, prolong waiting times, and prevent timely transplantation for many patients (3). In recent years, advances in immunomodulatory protocols and tolerance induction protocols have shown promise in mitigating immune rejection and expanding the donor pool. Tailored immunosuppressive regimen based on individual immunological profiles are emerging as key components of precision medicine, with the goal of optimizing graft survival and minimizing systemic toxicity (4).

Despite the scarcity of donor organ underscores the urgent need for alternative and innovative therapeutic strategies. In this context, regenerative medicine has emerged as a dynamic and transformative field (1, 5, 6).

Leveraging stem cell biology, tissue engineering, and bio-fabrication, regenerative medicine seeks to create renal tissues capable of restoring kidney function. Induced pluripotent stem cells (iPSCs) and adult stem cells serve as renewable sources of progenitor cells, while scaffold-based systems provide supportive environments that replicate native tissue architecture, supporting growth and repair (7).

Among the most groundbreaking developments are kidney organoids, miniaturized, lab-grown structures that recapitulate aspects of nephrogenesis. Numerous preclinical studies have investigated the transplantation of iPSC-derived kidney organoids into animal models, offering insight into their viability and partial functionality *in vivo* (8, 9). These examinations have yielded valuable insights into the feasibility and potential challenges associated with integrating organoids into host organisms (8, 10, 11). Complementary advances in bioengineering technologies, such as scaffold decellularization/recellularization, 3D bioprinting, interspecies blastocyst complementation, and progenitor cell replacement, are broadening the spectrum of approaches aimed at replicating the complex architecture and function of native kidney (3).

Nonetheless, the generation of fully transplantable, functional kidneys remains a formidable challenge. Critical barriers include precise control over cellular differentiation, tissue organization, and vascularization (12, 13), as well as translational concerns related to safety, efficacy, and regulatory oversight (8–12). Ethical considerations surrounding advanced biotechnologies further complicate the clinical trajectory (14). Despite these hurdles, sustained research efforts continue to explore both regenerative therapies and novel transplantation strategies. Genome editing and xenotransplantation, for example, are being employed to overcome limitations related to immune compatibility and donor scarcity. The integration of these innovative modalities holds great promise for reshaping the landscape of kidney replacement therapy (15, 16).

This scoping review aims to synthesize, the current evidence base surrounding regenerative and bioengineering approaches to ESRD. critically evaluating their scientific rationale, preclinical validation, and translational potential.

Specifically, it seeks to answer the following questions:

− What is the current and potential role of regenerative medicine in the treatment of ESRD?
− What preliminary results support its efficacy in addressing renal failure?
− What methodologies and experimental models have been explored to date?
− Are there any validated, evidence-based bioengineering applications for renal replacement?
− Which aspects of regenerative medicine are most debated or promising in the current scientific literature?

Through a rigorous and comprehensive analysis, this review intends to highlight opportunities, challenges, and future directions in the pursuit of regenerative and personalized therapeutic solutions for end-stage renal disease.

## Methods

### Protocol and registration

Our protocol was drafted using the Preferred Reporting Items for Systematic Reviews and Meta-analysis Protocols (PRISMA-ScR), as revised by Tricco et al. (2). The final protocol was registered prospectively with the online repository Figshare (17) on 27 April 2024.

### Eligibility criteria

Inclusion and exclusion criteria were developed by the authors during several meetings and guided by the conceptual framework developed for this review.

Peer-reviewed journal papers published from 2004 to 2024 were included, reflecting two decades of significant advancement in the field of bioengineering (3). The proposed study covers documents published in any language and imposes no geographical limitations. Descriptive, observational, qualitative, narrative, systematic and mixed methods reviews prior to this work were included. Conversely, studies published in low-impact journals, editorials or letters were excluded.

Included articles covered a range of bioengineering techniques, such as organoid generation, scaffold development, 3D bioprinting, stem cells, artificial mitochondrial transfer, organ-on-a-chip, normothermic machine perfusion, xenotransplantation, interspecies progenitor replacement, and interspecies blastocyst complementation. Papers were excluded if they did not fit into the conceptual framework of the study, Reviews on wearable artificial kidneys, for example, were omitted for this reason.

The only organ of interest for this scoping review is the kidney, so all other organs were disregarded.

The specific disease examined is ESRD, hence articles concerning other pathologies were excluded.

### Information sources

To identify potentially relevant documents, the following bibliographic databases were searched from 2004 to March 2024: Medline, Scopus and Web of Science.

The search strategies were drafted by AV and PB and further refined through team discussion. The final search results were exported into EndNote, and duplicates were removed by the authors.

LA overhauled the protocol; GP and MM revised the manuscript and English language fluence; DI helped with data collection and screening; DDG and CG revised the manuscript; AB and GI guided the study design and checked all stages of manuscript drafting.

### Search strategy

The search strategy involved the following subject headings and keywords: “bioengineering”, “kidney”, “renal”, “bioprinting”, “organoids”, “scaffold” “regenerative”, “stem”, “normothermic machine perfusion”, “artificial mitochondrial transfer”, “xenotransplantation”, “interspecies blastocyst complementation” and “chip”. Electronic databases to be searched were Medline, Scopus and Web of Science.

References of all relevant studies were also checked to expand the database reviewed. Relevant articles were selected and included if the inclusion criteria were met.

The search strategies employed by each library that was explored can be found in Appendix 1.

### Selection of sources of evidence

To enhance uniformity each reviewer examined all the publications, deliberated on the findings, and revised the screening and data extraction guidelines before commencing the review process. Four reviewers, collaborating in pairs, systematically assessed the titles, abstracts, and subsequently the full text of all publications identified through our search for pertinent literature. Any discrepancies in study selection and data extraction were addressed through consensus and, if necessary, through discussion with other reviewers.

### Data charting process

Two reviewers collaboratively designed a data-charting form to identify the variables for extraction. Independently, they charted the data, engaged in discussions regarding their findings, and iteratively refined the data-charting form as part of an ongoing process.

### Data items

We extracted data on article characteristics based on the inclusion criteria summarized in **Table 1**. The search covered a 20-year timeframe, from January 2004 to March 2024, without restrictions on language or geographical origin. Eligible studies included descriptive, observational, qualitative, mixed-methods, narrative, and systematic reviews.

**Table 1:** Shows the number of articles for each analyzed technique.

Technique	Number of articles
Kidney Organoids	34
Scaffold	13
3D Bioprinting	16
Stem Cells	2
ATM	2
Xenotrasplantation	7
IBC	6
IPR	2
Organ-on-a-chip	55

We excluded editorials, letters, and publications from non-impact journals.

To ensure a comprehensive overview of regenerative approaches, studies investigating normothermic machine perfusion were also included. Only articles explicitly proposing interventions for ESRD were retained for analysis.

### Synthesis of results

We organized the studies based on the technique they examined and provided summaries for each group including the year of publication, language, study designs, measures used, and overarching findings. If a systematic or narrative review was detected, we tallied the number of studies meeting our inclusion criteria within the review and noted any studies overlooked by our search.

### Author contributions

PB and AV were responsible for the manuscript’s initial drafting, subsequent revisions, and research methodology development. DDG, LA, CG, DI, GP, and GC provided feedback on the manuscript. DI served as the corresponding author. GI and AB were responsible for coordinating and supervising the study.

## Results

Our databases search, as outlined in the methods section, identified 5,869 scientific papers. Following removal of 2,625. 3,244 unique articles underwent title and abstract screening. Of these, 2,801 articles were excluded based on the predefined inclusion criteria.

Of the 443 articles selected for full-text review, 320 were further excluded as they did not align with the inclusion criteria or address the research questions pertinent to this study.

**Figure 1:**
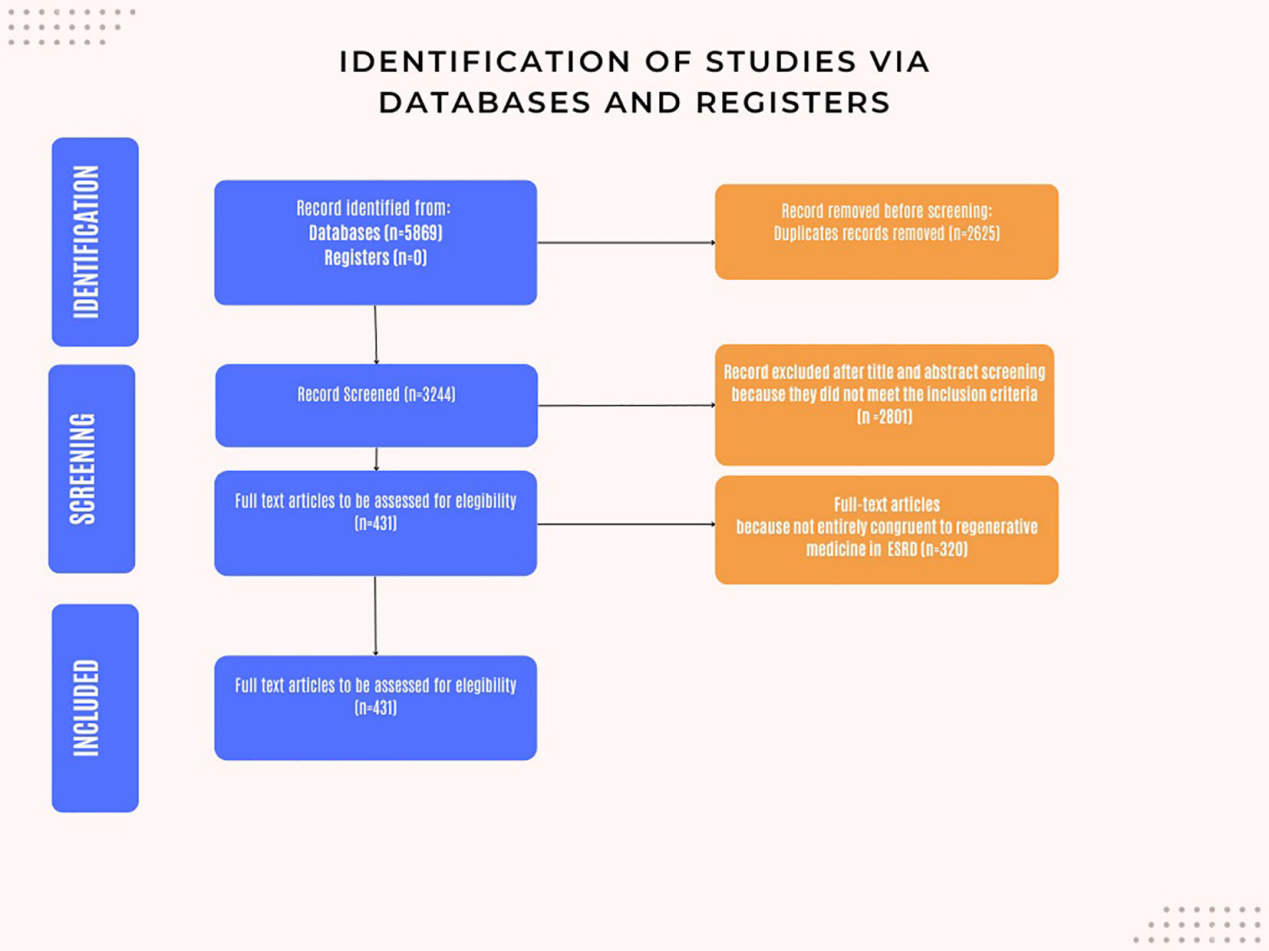
Flow chart: Identification of the studies via databases and registers. The following is a brief exposition of the techniques analyzed in this review.

Consequently, 111 articles were selected for full-text review, of which 110 were in English language and 1 was in Mandarin (**Figure 1**).

### Stem cells

Stem cells are undifferentiated cells capable of self-renewal and differentiation into specialized cell types. They serve as a continuous source of differentiated cells, essential for the formation, maintenance, and repair of tissues and organs in both animals and plants (17).

**Figure 2:**
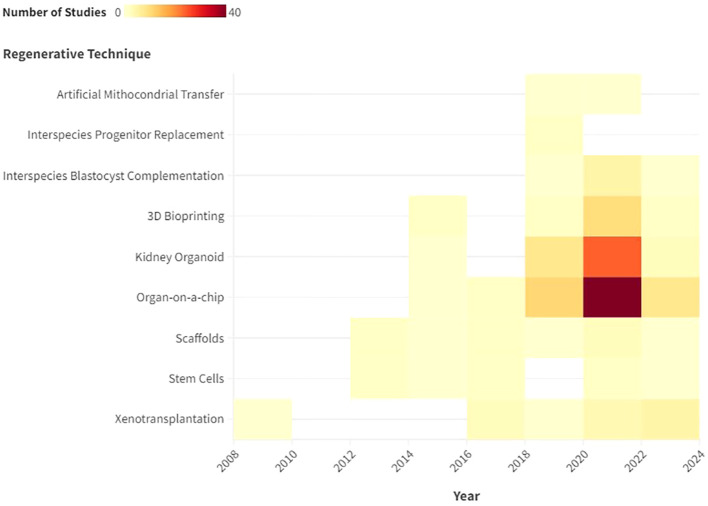
Description with heat map of the number of publications that met the inclusion criteria by year.

From 2013 to 2024, the number of scientific papers involving the use of stem cells and meeting the inclusion criteria remained stable, as the heat map shows (**Figure 2**). Two narrative reviews focusing on stem cell applications in renal regeneration were included.

### Kidney organoid

Kidney organoids are innovative *in vitro* renal models used in biomedical research that are produced from stem cells in 3D cultures. Stem cells are supplemented with nutrients and chemical signals like growth factors, which drive them to differentiate and self-organize. Organoids exhibit a remarkable degree of similarity to the native tissue in terms of cell type, morphology, and function (5). Organoids tend to be developed for the purpose of studying specific characteristics of the target organ that cannot be easily replicated in animal models or in 2D cultures (6). The articles selected for review address the topic of ischaemic-reperfusion injury. Since 2015, the number of studies addressing this topic has increased steadily. In 2022, 15 studies were published on the renal organoids that met the inclusion criteria. The final selection included 26 narrative reviews, seven original studies and 1 evidence review.,

### Scaffolds

Scaffolds represent important components for tissue engineering, providing structural support for cells to attach, grow, migrate and differentiate *in vitro* and *in vivo*. They provide shape and mechanical stability to the tissue, while maintaining structure and stiffness in the engineered construct (4, 8). Regarding the utilization of scaffolds, the focus of the extant literature has remained largely consistent over recent years. The inaugural study selected for review was published in 2013, after which one article was published approximately every year, amounting to a total of 13 articles submitted, consisting of 8 narrative reviews and 5 original studies.

### 3D bioprinting

3D Bioprinting is an advanced manufacturing process that creates complex biomedical structures using a layer-by-layer deposition of cells, biological molecules, and biomaterials. The most important motivation behind the development of 3D bioprinting is the limited availability of biological structures required for the rehabilitation of lost organs and tissues. The aim of the process is to provide a suitable alternative to tissue implants and animal testing procedures during research on diseases and the development of treatments (9). Currently, the use of 3D bioprinting is narrowed to tissue formation for the estimation of drug efficacy, but 3D bioprinting has great scope in its use for replacing lost and failed organs in patients.

3D bioprinting is becoming increasingly important. The first study selected for review was published in 2015, after which there was a notable increase in the number of scientific publications on this biological technique. In 2022, seven studies were published that met the inclusion criteria of this scoping review. From the first study selected in 2015, to the last in March 2024, the authors collected 16 studies, including 14 narrative reviews and 4 original studies.

### Organ-on-a-chip

Organs-on-chips, also known as micro-physiological systems (MPS), are bioengineered microsystems capable of recreating aspects of human organ physiology and function. The technology was developed as an alternative method for modelling human diseases *in vitro* to accelerate new drug development and advance personalized medicine, therefore replacing animal models that old limited human applicability (15). This technique is particularly effective in recreating shear forces and flows that can be found in real organs, and that cannot be reproduced using techniques such as organoids.

**Figure 3:**
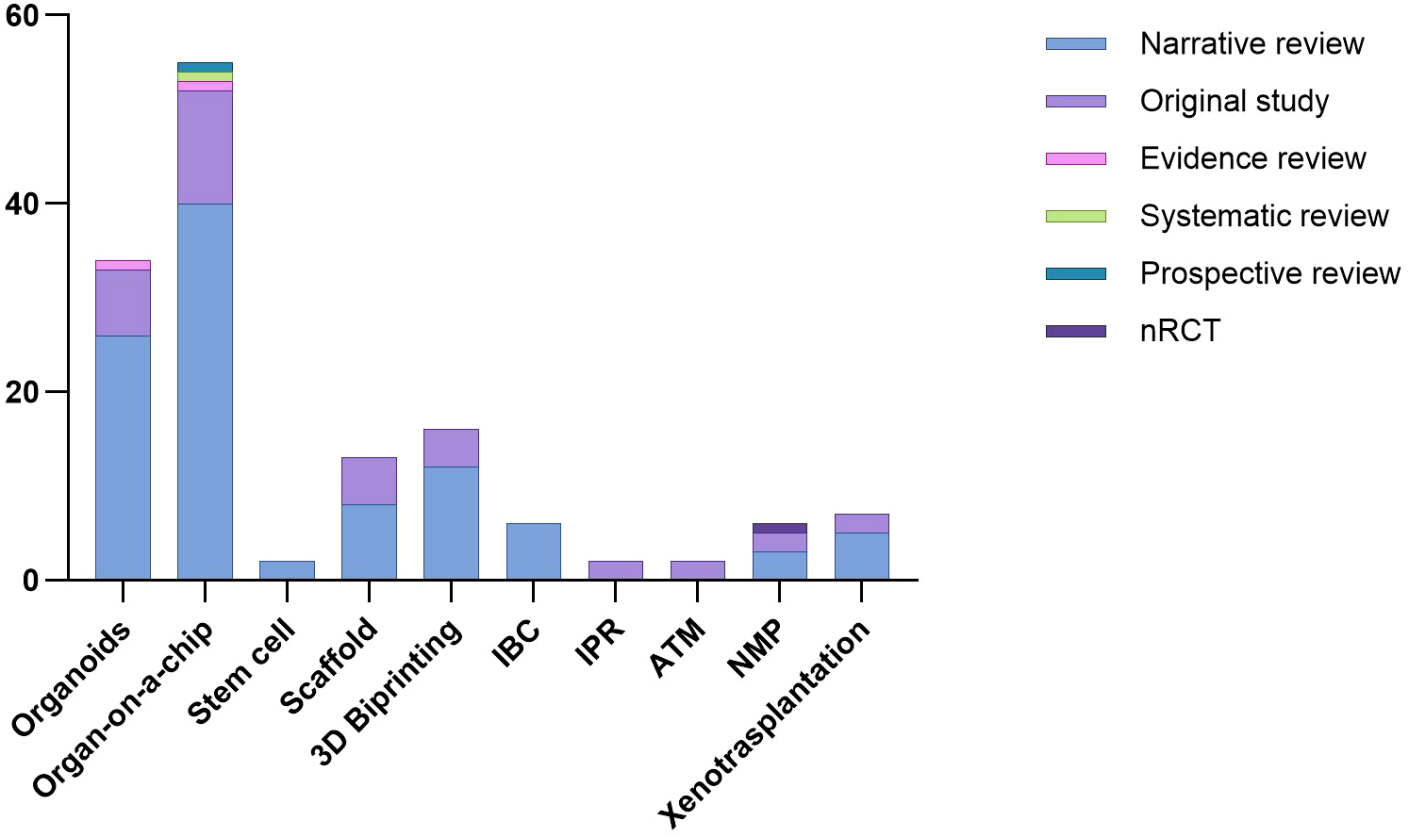
Trends in the number and type of studies according to the bioengineering technique used.

This was the most extensively documented technique, with 58 included studies 55 narrative reviews, 12 original studies, 1 evidence, 1 systematic and 1 prospective review and a publication peak between 2020 and 2022 (**Figure 3**).

### Interspecies blastocyst complementation

IBC is a strategy that enables the generation of interspecies chimeras through blastocyst complementation, in which heterologous pluripotent stem cells (PSCs) are injected into the blastocyst of an animal that has been genetically manipulated to lack a specific organ. The aim of IBC is to generate organs in animals and transplant them into humans (7).

There has been increasing interest in this technique in the last six years. The first study was published in 2018, and since then, a further six studies meeting the inclusion criteria have been published. All of them were narrative review.

### Interspecies progenitor replacement

The strategy behind IPR involves injecting donor cells during the kidney formation stage rather than the blastocyst stage. Unlike blastocyst complementation, this method can avert the migration and distribution of injected cells to off-target organs (7).

A total of two original studies were selected that pertain to this technique over the two-year period spanning 2018 to 2020.

### Artificial mitochondrial transfer

Mitochondrial transplantation is a breakthrough therapeutic strategy that aims to replace native mitochondria damaged by ischemia-reperfusion injury by injecting viable respirable mitochondria isolated from non-ischaemic tissue from the same subject (13, 14).

This technique has not been widely described in the existing literature. In fact, only two experimental studies have been identified that met the inclusion criteria.

### Normothermic machine perfusion

Normothermic machine perfusion (NMP) is a novel method employing cardiopulmonary bypass technology alongside extracorporeal membrane oxygenation to perfuse kidneys using a warmed and oxygenated red-cell-based plasma-free solution (10, 11). This technique sustains organs in a state close to normal physiological conditions, facilitating *ex vivo* functional restoration and testing. Furthermore, NMP offers metabolic resuscitation by replenishing depleted ATP levels resulting from a combination of warm and cold ischemia.

Regarding *ex situ* normothermic perfusion, a considerable number of articles published between 2015 and the present have addressed the treatment of terminal renal failure. Even though these studies propose *ex situ* normothermic perfusion as an alternative to standardized graft reconditioning techniques, most of these were excluded at the screening stage due to the lack of significant evidence.

Recent studies have shown that cell therapy can be successfully delivered directly to donor kidneys using an isolated *ex vivo* perfusion system. Using stem cells during normothermic machine perfusion (NMP) has demonstrated improvements in clinically relevant parameters and reductions in injury and pro-inflammatory biomarkers. These positive effects may be due to changes in circulating cytokines or the release of soluble anti-inflammatory mediators. NMP is emerging as a promising method for delivering cell therapy, offering a new way to treat organs before transplantation and potentially reducing ischemia-reperfusion injury (IRI). This innovative approach could transform transplantation practices by making more organs viable for transplant, thereby shortening waiting lists and providing new hope for patients with renal failure. Further clinical trials are needed to evaluate the effectiveness and safety of this delivery method in clinical settings (18, 19).

As illustrated by the heat map, the number of normothermic renal perfusion studies that met the inclusion criteria remained stable from 2016 to 2024. These included 3 narrative review, 2 original studies, and 1 non-randomized controlled trial (nRCT).

### Xenotransplantation

Xenotransplantation of the kidney is the scientific practice of transplanting a kidney from one species to another, typically involving the transfer of a kidney from a non-human donor, such as a pig, to a human recipient, with the aim of addressing organ shortage and improving transplant outcomes (3). Renal xenotransplantation is gaining increasing importance in the field of regenerative medicine. However, the high cost and high specialization makes this technique feasible for study purposes only in very few centers worldwide. Nevertheless, we have seen an increase in interest in the literature over the last 6–8 years. In fact, 7 articles were included in the study. Of them, 5were narrative reviews and 2 were original studies (**Table 2**).

**Table 2:** Overview of all regenerative medicine techniques explored for the treatment of end-stage renal failure (3, 6, 20–28).

Technique	Advantages	Disadvantage	Recent updated findings (*in vivo* or *in vitro*)	Challenging points in individual strategies
Kidney Organoids	Disease modelling Drug testing Personalized Medicine Developmental Studies Cytotoxicity studies Ethical Alternatives	Complexity Variability Maturation Limited lifespan Cost	Vascularization Genome editing Bioengineering Microfluidic Integration Transcriptomics	Reproducible culture condition *In vivo* like microenvironment
Scaffold	Regenerating damaged or lost structures without the need for permanent implants Biocompatibility Biodegradable and Bioresorbable Structural and biomechanical stability	Thrombosis Number of cells initially required depending on the organ type Culture system characteristics and stability Cost Choosing the correct perfusate Oxygen and nutrient supply	Whole-organ grafts produce to replace or support function *in vivo* for few hours	*In vivo* graft viability and function of mid-term (days to weeks) and long-term (months) in healthy animal models
3D bioprinting	Construction of micro fluidic devices Generation and transplantation of multiple tissue types	Choosing the right bio ink Placing cells at the correct distance Cost Operator training Bioreactor	Generation of ‘mini-tissue’ Bioprinting of 3D structure of human renal proximal tubules *In situ* bioprinting	Bioprinting of complex and large 3D organ Incorporation of a vascular network Maintaining high cell viability Application of machine learning
NMP	Returns new quality parameters, such as urea and creatinine levels, or electrolytes and acid-base balance	No evidence of superiority over current techniques (hypothermic perfusion, static cold storage)	Implants of totipotent stem cells during renal perfusion improve urinary output	Improving the outcome of transplanted patients, as well as providing information on graft transplantability
Stem Cells	Regenerative potential Application in tissue engineering field Immune compatibility Ethical alternatives	Need to elucidate underlying mechanisms before clinical application	Mesenchymal stem cells treatment during exsanguinous metabolic support reported its potential to accelerate the regeneration	Elucidate the mechanisms underlying their paracrine effects
ATM	Potentially minimally invasive approach	Scarce evidence, limited number of preclinical studies	Mitochondrial nesting would appear to support renal function	Making the technique reproducible on a large scale
Xenotrasplantation	Creation of customized organs, reducing ischemia times and waiting lists. No immunosuppression.	Ethical issues	Improved outcomes of renal xenograft implantation in humans	Making the technique reproducible on a large scale
IBC	Creation of customized organs, reducing ischemia times and waiting lists. No immunosuppression.	Ethical issues; Hyperacute rejection of the host cells; Creation of hybrid animal-human.	Culture of human-monkey embryos was completed at 20 days after fertilization	it is impossible to control the proportion of chimeric cells distributed within the host animal
IPR	Creation of customized organs, reducing ischemia times and waiting lists. No immunosuppression. Avoid the distribution of injected cells to off-target organs	Ethical issues; Hyperacute rejection of the host cells; Creation of hybrid animal-human.	Nephrons progenitor cells in mice were simultaneously replaced with the corresponding cells from rats	Replace all cell lineages at the same time
Organs-on-a-chip	Disease studying Drug development Personalized Medicine Developmental Studies Ethical Alternative	Standardization of the manufacturing process Lack of protocols Cost	Can provide a complex and physiological functions Capability to regulate fluid movements	Developing a blood mimetic universal medium Mimic the interaction between organs

## Discussion

### The role of regenerative medicine in ESRD treatment

**Figure 4:**
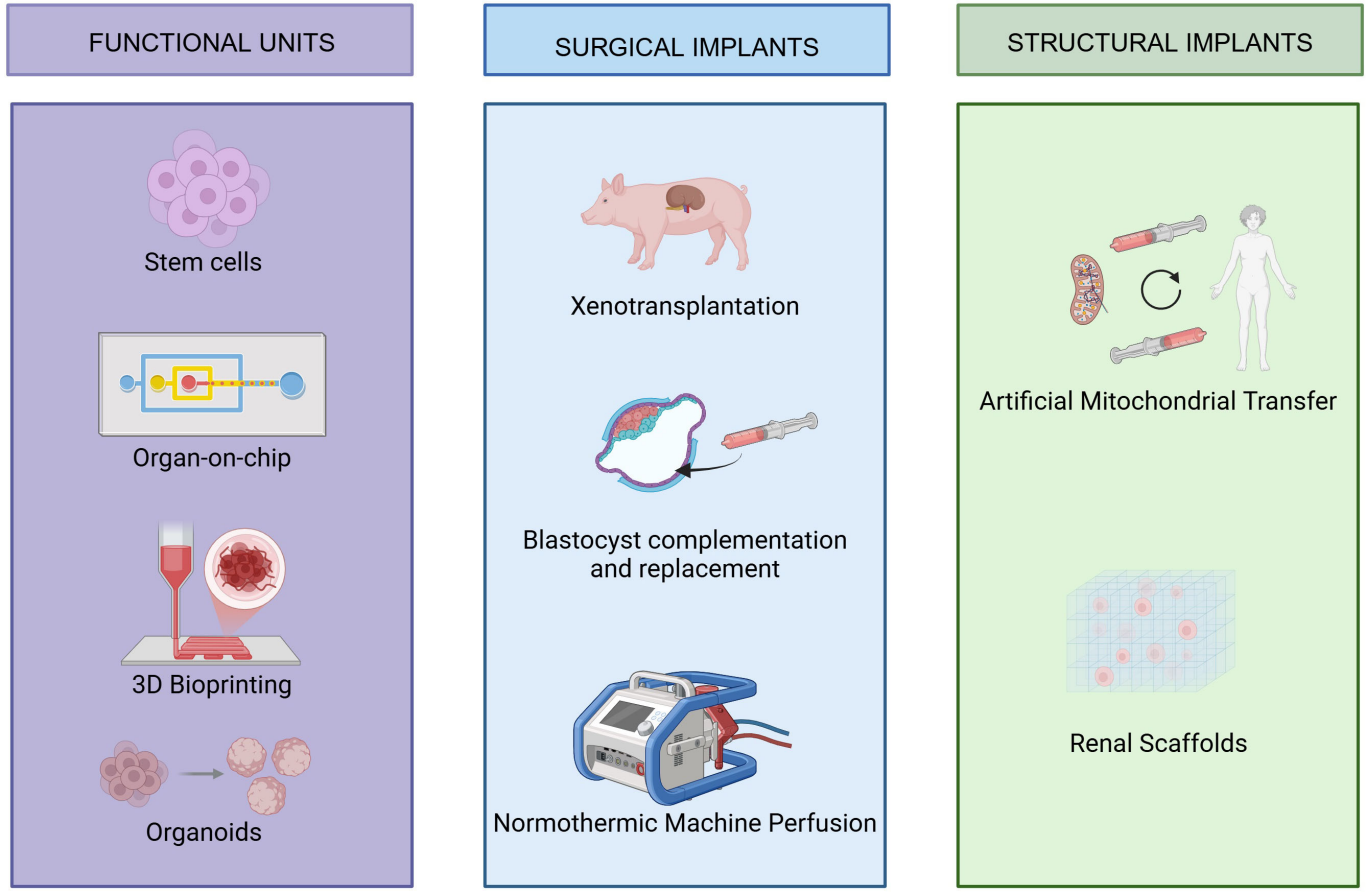
Current regenerative medicine strategies to face ERSD.

Regenerative medicine is emerging as a promising frontier in the treatment of end-stage renal disease (ESRD), offering alternatives to dialysis and transplantation. Notably, kidney organoids and organ-on-a-chip systems are at the forefront. Organoids mimic nephron-like structures derived from stem cells, while microfluidic chips replicate renal physiology under controlled conditions. Together, they provide powerful platforms for disease modeling, therapeutic testing, and advancing bioengineered kidney development (3, 13) (**Figure 4**).

### Preliminary results and current evidence

Renal organoids and kidney on-a-chip systems hae gained momentum due to their effectiveness in drug screening, disease modeling, and studying renal development.

Multiple cell lines have been utilized, including MDCK, opossum, porcine, and especially human proximal tubular epithelial cells (hPTECs). Yet, limitations in cell expansion and phenotypic stability restrict their long-term utility (18, 19).

A milestone in the field is the glomerulus-on-a-chip, developed using iPSC-derived podocytes (29, 30). This model successfully mimics glomerular filtration and injury responses, including adriamycin-induced albuminuria and autoantibody-mediated damage. It also enhances understanding of diseases like diabetic nephropathy through the study of pathways such as Notch signaling in podocyte regeneration.

These technologies offer critical insight into renal pathophysiology and are key steps toward functional artificial kidneys.

### Studies on various approaches

While organoids and chip-based models show strong early promise, other regenerative strategies—including 3D bioprinting, xenotransplantation, and normothermic machine perfusion—are at earlier stages of validation (3, 10, 15, 29–31).

The current literature is rich in narrative reviews but lacks robust experimental and clinical studies (15, 30).

Organoids are well-characterized *in vitro*, yet clinical translation is limited. Similarly, 3D bioprinting has captured interest in its potential in organ fabrication, but few studies address its long-term integration and functionality *in vivo*.

Xenotransplantation remains hindered by immunologic and ethical concerns, and while normothermic perfusion is promising, standardization and expanded trials are necessary for broader clinical adoption.

### Evidence-based applications of normothermic machine perfusion and xenotransplantation

Among the most clinically advanced strategies, normothermic machine perfusion (NMP) has demonstrated improved organ preservation and graft function. For instance, prolonged Normothermic Ex Vivo Kidney Perfusion (NEVKP) in porcine models significantly reduced injury markers and enhanced metabolic activity compared to static cold storage (SCS), while shorter NEVKP durations showed minimal benefits.

Nowadays, emerging studies on NMP are implementing durability and propose new perfusate compositions. Unfortunately, most of the reviews analyzed on normothermic highlighted very high costs in terms of economics, materials and logistics, compared to results that are not superior to traditional methods like hypothermic perfusion machine or ice.

Xenotransplantation, particularly pig-to-primate kidney grafts, has progressed through the use of genetically modified donors and targeted immunosuppression (e.g., anti-CD40/anti-CD154 antibodies). Graft survival now exceeds 400 days in some models, although complications like proteinuria and antibody-mediated rejection persist. Future advances will rely on deeper genetic editing and immune modulation to ensure long-term graft viability (32).

Despite encouraging results, both approaches face ethical and regulatory challenges that warrant cautious progression.

### Key discussion points and recommendations for future research

The future of regenerative medicine in the treatment of ESRD will be shaped by several critical considerations (3, 14). Among the most pressing are the ethical and logistical challenges associated with implementing emerging technologies, the need to ensure that advanced platforms are scalable and suitable for large-scale production, and the importance of validating their long-term safety and efficacy through rigorous testing.

To address these challenges, future research should prioritize experimental trials that evaluate the clinical potential of kidney organoids and organ-on-a-chip systems. In addition, *in vivo* studies are needed to assess the functionality and integration of 3D bioprinted renal tissues. Further refinement and standardization of xenotransplantation and NMP protocols will also be essential to advance these approaches toward clinical use.

Ultimately, the successful translation of these innovations into real-world treatments will depend on strong interdisciplinary collaboration, robust ethical oversight, and sustained investment in research and development.

## Conclusions

The potential of regenerative medicine for the treatment of ESRD is becoming increasingly evident. A comprehensive review of the current scientific literature reveals a significant number of articles exploring the use of kidney organoids and organ-on-a-chip technologies, which are gaining prominence in the field of regenerative medicine. In contrast, other techniques, despite their potential, appear to be less of a focus, despite offering a diverse range of applications. Organoids and organ-on-a-chip systems have demonstrated potential in drug screening, disease modeling, and understanding renal development. These advancements could support the transition treatment from traditional dialysis and transplantation to regenerative approaches that restore kidney function.

Despite progress, most studies are narrative reviews, highlighting a need for more experimental and prospective research to validate these technologies.

3D bioprinting, xenotransplantation, and normothermic machine perfusion show promise in the areas examined by this review. While some have already been tested in humans, further implementation is necessary for them to become a valuable clinical tool. Future research should focus on empirical validation, interdisciplinary collaboration, and ethical considerations to advance these technologies into clinical practice.
